# Pandemic GII.4 Sydney and Epidemic GII.17 Kawasaki308 Noroviruses Display Distinct Specificities for Histo-Blood Group Antigens Leading to Different Transmission Vector Dynamics in Pacific Oysters

**DOI:** 10.3389/fmicb.2018.02826

**Published:** 2018-11-27

**Authors:** Vasily Morozov, Franz-Georg Hanisch, K. Mathias Wegner, Horst Schroten

**Affiliations:** ^1^Pediatric Infectious Diseases Unit, University Children’s Hospital Mannheim, Medical Faculty Mannheim, Heidelberg University, Mannheim, Germany; ^2^Institute of Biochemistry II, Medical Faculty, University of Cologne, Cologne, Germany; ^3^Coastal Ecology, Wadden Sea Station Sylt, Alfred Wegener Institute – Helmholtz Centre for Polar and Marine Research, List auf Sylt, Germany

**Keywords:** norovirus, histo-blood group antigens (HBGA), oyster, norovirus outbreak, food pathogens, norovirus transmission, food safety

## Abstract

Noroviruses are the major cause of foodborne outbreaks of acute gastroenteritis, which are often linked to raw oyster consumption. Previous studies have suggested histo-blood group antigens (HBGA)-like structures in the oyster tissues as ligands for norovirus binding and persistence. To better understand how oysters function as vectors for the most common human noroviruses, we first tested the ability of the norovirus strains GI.1 West Chester, the pandemic GII.4 Sydney, and the epidemic GII.17 Kawasaki308 strains to interact with oyster tissues. Secondly, we explored how the HBGA preferences of these strains can affect their persistence in oyster tissues. We found limited HBGA expression in oyster tissues. HBGAs of A and H type 1 were present in the digestive tissues and palps of the Pacific oyster *Crassostrea gigas*, while the gills and mantle lacked any HBGA structures. By using Virus-like particles (VLPs), which are antigenically and morphologically similar to native virions, we were able to demonstrate that VLPs of GI.1 West Chester norovirus reacted with the digestive tissues and palps. Despite of the lack of HBGA expression in mantle, dominant GII.4 Sydney strain readily bound to all the oyster tissues, including the digestive tissues, gills, palps, and mantle. In contrast, no binding of the epidemic GII.17 Kawasaki308 VLPs to any of the investigated oyster tissues was observed. In synthetic HBGA and saliva-binding assays, GI.1 reacted with A type, H type, and Le^b^ (Lewis b) HBGAs. GII.4 Sydney VLPs showed a broad binding pattern and interacted with various HBGA types. Compared to GI.1 and GII.4 VLPs, the GII.17 Kawasaki308 VLPs only weakly associated with long-chain saccharides containing A type, B type, H type, and Le^b^ blood group epitopes. Our findings indicate that GI.1 and GII.4 noroviruses are likely to be concentrated in oysters, by binding to HBGA-like glycans, and therefore potentially leading to increased long term transmission. In regards to the GII.17 Kawasaki308 strain, we suggest that oysters can only function as short term transmission vector in periods of high environmental virus concentrations.

## Introduction

Noroviruses are non-enveloped single stranded RNA viruses of the *Caliciviridae* family. According to the most recent classification, there are seven genogroups (GI- GVII) in the norovirus genus (Vinjé, 2015). Noroviruses of the GI and GII genogroups have been detected in humans. However, most norovirus infections over the past decade have been caused by genogroup II genotype 4 (GII.4) noroviruses. The GII.4 variants are responsible for six pandemics and for the majority of sporadic outbreaks worldwide (Noel et al., 1999; Widdowson et al., 2004; Bull et al., 2006; Eden et al., 2010, 2014; Yen et al., 2011). Recently a novel GII.17 strain has emerged in Asia, causing an alarming number of infections and gradually replacing GII.4 strains (Chan et al., 2015a; Zhang et al., 2015; Koromyslova et al., 2017).

Norovirus are highly infectious, with as few as ten particles being able to cause disease (Teunis et al., 2008). Viral transmission occurs either via direct person-to-person contact or indirectly through contaminated water or food. In fact, noroviruses are considered one of the most common cause of foodborne infections associated with gastroenteritis outbreaks (Atmar and Estes, 2006; Moore et al., 2015; de Graaf et al., 2016). Many food-related disease outbreaks of acute gastroenteritis caused by noroviruses are associated with oyster consumption. Oysters are filter-feeding epibenthic bivalves, which filter up to 19 liters of water per hour and gram bodyweight (Jørgensen, 1996), and have been found to accumulate different pathogens from sea water. Oysters become contaminated with norovirus by exposure to discharging municipal water supply (Lees, 2000) and their raw consumption often results in outbreaks of acute gastroenteritis (Ang, 1998; Doré et al., 2010; Westrell et al., 2010).

It has been suggested that noroviruses are bound by oyster tissues through specific carbohydrate-mediated interactions, which involve the norovirus capsid protein (viral protein 1, VP1) and human-like histo-blood group antigens (HBGA). Human HBGAs are carbohydrate epitopes at the terminal end of O-glycans on glycoproteins and of glycolipids on the surface of red blood cells, the mucosal epithelium of the gastrointestinal, and the respiratory and the genitourinary tracts. Specific HBGAs, similar to blood group A type and H type, have also been identified in the digestive tracts of different oyster species (Tian et al., 2007; Ma et al., 2017).

The human noroviruses interact with HBGAs and oyster tissues in a strain-dependent manner. The GI.1 Norwalk noroviruses exhibit strong preferences toward A-type and H-type glycans and bind to the oyster digestive tissues (Le Guyader et al., 2006; Tian et al., 2006, 2007; Maalouf et al., 2010, 2011). Importantly, the GI.1 Norwalk VLP binding correlates with the expression of A-type HBGAs in the oyster digestive tract (Tian et al., 2007). Lastly, both GI.1 Norwalk VLPs and GI.1 virions from a stool sample were shown to be efficiently bioaccumulated by *Crassostrea gigas* (Maalouf et al., 2010, 2011). In contrast, the GII.4 Houston norovirus readily interacts with the digestive tissues, gills, and mantle tissue extracts through sialylated carbohydrates. Despite this, only low levels of GII.4 Houston norovirus VLPs were bioaccumulated by *Crassostrea gigas*, which was suggested to be due to an unknown degradation mechanism (Maalouf et al., 2011).

The dominant GII.4 Sydney 2012 noroviruses are most abundant among GII strains detected in samples from the oyster-related outbreaks (Yu et al., 2015). Likewise, recent reports have found epidemic GII.17 noroviruses in oysters collected from the coasts of Italy, Japan, and Korea (Shin et al., 2013; Pu et al., 2016; La Rosa et al., 2017). Moreover, Rasmussen et al. directly linked a series of acute gastroenteritis infections with the consumption of GII.17-contaminated oysters (Rasmussen et al., 2016). In this work, we aimed at gaining a better understanding of how GII.4 Sydney and GII.17 Kawasaki308 strains are potentially transmitted to humans via oysters. To this end, we expressed the human norovirus capsid protein in insect cells. The expression resulted in the formation of VLPs, which are antigenically and morphologically analogous to native virions (Harrington et al., 2004; Hansman et al., 2006; Bok et al., 2009; Lindesmith et al., 2011, 2012, 2013; Ajami et al., 2012). Next, we measured oyster-tissue specificity of the GII.4 Sydney and GII.17 Kawasaki308 norovirus VLPs. We explored how binding preferences of these strains toward specific HBGAs can affect their persistence in oyster tissues. We compared the behavior of GII.4 and GII.17 noroviruses with the GI.1 norovirus, which is commonly found in oysters (Yu et al., 2015). The combination of these approaches will thus allow us to make specific predictions of the expected epidemiology of these norovirus strains.

## Materials and Methods

### Reagents

We examined VLP binding to multivalent-HBGAs conjugates purchased from GlycoTech (Maryland, United States). They included Le^y^-PAA-biotin (01-043), Le^b^-PAA-biotin (01-042), H type1(tri)-PAA-biotin (01-037), Le^x^-PAA-biotin (01-036), Le^a^-PAA-biotin (01-035), H type 2 (tri)-PAA-biotin (01-034), Blood type A (tri)-PAA-biotin (01-032), Blood type B (tri)-PAA-biotin (01-033). The following monoclonal antibodies (MAbs) were purchased for the HBGA phenotyping: A type antibodies (clone Birma-1, Medtro GmbH), B type antibodies (clone LB-2, Medtro GmbH), H type 1 antibodies (clone 17-206, Invitrogen), Lewis a antibody (clone 7LE, ThermoFisher), Lewis b antibody (clone 25LE, Abnova), Lewis x antibody (clone 73–30, TCI chemicals), Lewis y antibody (clone H18A, TCI chemicals). Porcine gastric mucin type III (PGM) was purchased from Sigma-Aldrich (M2378). Goat α-mice IgG HRP conjugated antibodies were purchased from ThermoFisher (62-6520).

### Norovirus Virus-Like Particles (VLPs)

The GI.1 West Chester (2001, AY502016), GII.4 Sydney (X459908), GII.17 Kawasaki308 (2015, LC037415) VLPs were produced in *Spodoptera frugiperda* (Sf9) cells using baculovirus expression system as described previously (Koromyslova et al., 2017). In short, the recombinant VP1 bacmids were transfected into the Sf9 cells using Effectene (Qiagen). After 5 days of incubations at 27°C the cells were harvested, centrifuged, and the supernatant containing baculovirus was used to infect high five (H5) insect cells. The H5 cells were incubated at 27°C for 6 days. At 6 days post infection, the supernatant containing secreted VLPs was collected. The VLPs were concentrated by ultra-centrifugation at 35000 rpm (SW55 Ti rotor, Beckman Coulter) for 2 h at 4°C and purified by the CsCl equilibrium gradient ultracentrifugation at 35000 rpm (SW55 Ti rotor, Beckman Coulter) for 18 h at 4°C. The morphology of the VLPs was examined by electron microscopy.

### Oyster and Saliva Samples

All experiments were performed with the Pacific oysters *Crassostrea gigas*. The oysters were collected from an uncontaminated area on Sylt, North Sea, Germany, 54.937962° N, 8.313825° E, and shipped the same day (within 24 h) on ice to the laboratory in Mannheim. Upon arrival, the oysters were immediately dissected and the digestive tissues, mantles, gills, and pulps were collected from five to six different oysters. The tissues were homogenized in phosphate-buffered saline (PBS) pH 7.4, boiled for 10 min at 95°C and centrifuged at 8000 rpm for 5 min (Mikro 200, Hettich). The supernatants were collected and used for ELISA experiments after the protein concentration was measured with the Bio-Rad protein assay kit. The tissue homogenate was tested for the presence of preexisting GI or GII norovirus contamination by a GeneXpert commercial assay according to the manufacturer’s instructions (Cepheid Inc., Sunnyvale, CA, United States) (data not shown). The oysters which were artificially exposed to norovirus-containing stool samples were used as a positive control.

Saliva samples collected from 17 healthy adult persons were boiled for 10 min at 95°C and centrifuged at 8000 rpm for 5 min (Mikro 200, Hettich). Approval was obtained from the ethics committee of the Medical Faculty of Mannheim, Heidelberg University, #2017-528N-MA.

### Detection of VLPs Binding to Oyster Tissues, Saliva Samples, and Synthetic HBGAs

The binding of the GI.1 West Chester, the pandemic GII.4 Sydney, and the epidemic GII.17 Kawasaki308 norovirus VLPs to oyster tissues was measured in triplicate for each oyster (Koromyslova et al., 2017). Briefly, NuncMaxisorp plates were covered with the digestive tract, mantle, gill, and pulp tissue extracts at a concertation of 40 μg/ml for 1 h at 37°C. The plates were washed three times with PBS-Tween20 0.1% (PBS-T) and blocked with 5% bovine serum albumin (BSA) for 1 h at 37°C. Subsequently, a serial dilution of VLPs (0–20 μg/mml, PBS) was applied to the plate for 1 h at 37°C. Plates were washed and incubated for 1 h at 37°C with either α-VLP-His_6_ nanobodies (Nb85) for GI.1 VLPs, α-rabbit-polyclonal antibodies for GII.4 VLPs, or α-VLP-biotinylated nanobodies (Nb26) for GII.17 VLPs (Koromyslova and Hansman, 2017). After washing the plate, the secondary antibodies (horseradish peroxidase conjugated with α-His_6_-IgG, α-rabbit-IgG or streptavidin) were added. Finally, the washed plates were developed for 30 min at room temperature with o-phenylenediamine (OPD) and H_2_O_2_. The reaction was stopped after 30 min and absorbance was measured at 490 nm on a Tecan infinite M200 Multiwell reader (Tecan, Switzerland). Negative controls included a VLP-negative control and primary antibody-negative controls. PGM and secretor saliva samples, described below in the results, were used as positive controls.

For the synthetic HBGA assays, carbohydrate-polyacrylamide - biotin (HBGA_x_-PAA-biotin) conjugates were diluted to a final concentration of 20 μg/ml in PBS and applied on streptavidin-coated plates overnight at 4°C. The plates were blocked and a serial dilution of VLPs (0–20 μg/mml, PBS) was applied. The concentration of bound VLPs was detected as described above.

For the saliva binding assay, 100 μl of the 1:50 diluted saliva samples (each sample was measured in triplicate) was added to carbohydrate buffer pH 9.4, and were incubated overnight at 4°C, then washed and blocked. The plates were incubated with 10 μg/ml VLPs in PBS and the concentration of bound VLPs was detected as described above.

### Detection of HBGA Present in Saliva and Oyster Tissues

The HBGAs phenotypes expressed in the oyster tissues and saliva samples were determined using MAbs as previously described (Tian et al., 2007). Each incubation step was followed by a washing step (3X PBS-T). The oyster tissues extracts were coated on NuncMaxisorp plates overnight at 4°C. Plates were blocked with 5% BSA in PBS-T for 1 h at 37°C. 100 μl of 1:400 α-A type MAbs, 1:100 α-B type MAbs, 1:250 α-H type 1MAbs, 1:50 α-Lewis a type MAbs, 1:1800 α-Lewis b type MAbs, 1:800 α-Lewis x type MAbs, and 1:3200 α-Lewis y type MAbs were incubated for 1 h at 37°C. Goat α-mice IgG HRP conjugated Abs were used in the 1:800 – 1:3200 dilution range for 1 h at 37°C. The signal was developed with the OPD/H_2_O_2_ mixture for 30 min and the intesity was measured on the Tecan reader.

### Statistical Analysis

Means were compared by using the Student *t*-test, and a *P*-value of below 0.05 was considered as statistically significant (GraphPad Prisma Software, La Jolla, CA, United States).

## Results

### GI.1, GII.4, and GII.17 VLP Binding to Oyster Tissues

**FIGURE 1:**
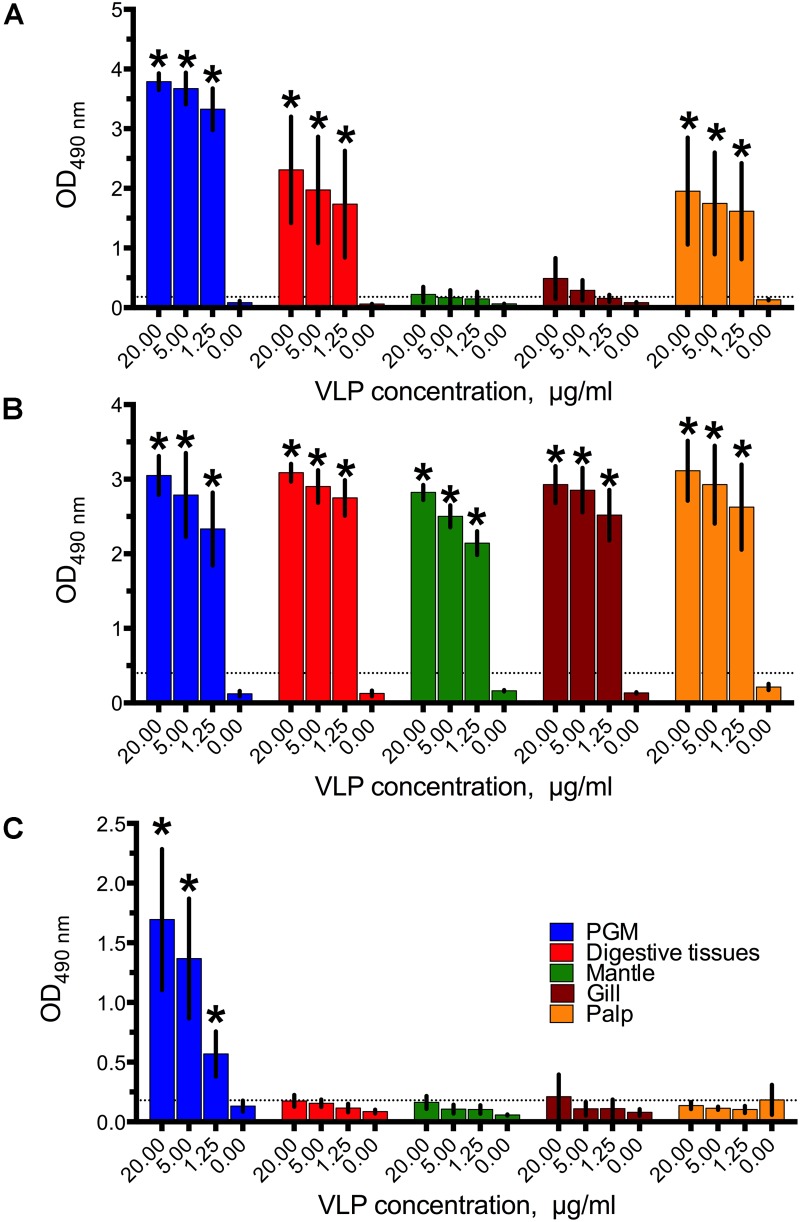
The binding of **(A)** GI.1West Chester VLPs, **(B)** GII.4 Sydney VLPs, **(C)** and GII.17 Kawasaki308 VLPs to digestive tissues, gills, mantle, and palp tissue extracts. All data are given as mean from five-six oysters measured separately ± standard deviations (SD), Asterisks indicate statistically significant differences (comparison to PBS control, *p* < 0.05).

GI.1 West Chester VLPs bound to the digestive tissues were easily detected even at the lowest concentration of 1.25 μg/ml, while no binding to mantle and gills was observed (Figure 1A). In contrast to previous studies (Maalouf et al., 2011) which used a different GI.1 strain, GI.1 West Chester VLPs also bound to palp tissue extracts. However, the binding was expected since A type HBGAs are present in palps (Figure 3A). This A type HBGA-dependency was indirectly confirmed by high standard deviations, which match the different levels of A type HBGA expression in the various oyster samples (Supplementary Figure S1).

Similar to the less prevalent GII.4 variant (Maalouf et al., 2011), the dominant GII.4 Sydney VLPs showed a strong binding efficiency to all tissues tested, even at the lowest VLP concentration (Figure 1B).

Surprisingly, no binding of the epidemic GII.17 Kawasaki308 VLPs to any of the oyster tissues was observed (Figure 1C). Although we obtained signals in the PGM coated wells (i.e., positive controls), all of the GII.17 VLPs assayed at a concentration of 20–1.25 μg/ml produced readings below the detection limit. The assay was repeated several times in parallel with the GI.1 and GII.4 binding experiments using the same tissue extracts. Additionally, testing higher VLP concentrations of 80 μg/ml, different coating procedures with higher concentrations of tissue extracts, and longer incubation times had no effect on the GII.17 VLP binding (Supplementary Figure S2). To exclude any seasonal factors which could have contributed to the GII.17 VLP binding, the assays were also conducted with oysters collected in December and June. However, these binding assays also resulted in qualitatively similar negative results (Supplementary Figure S3).

### Expression of HBGA in Oyster Tissues

**FIGURE 2:**
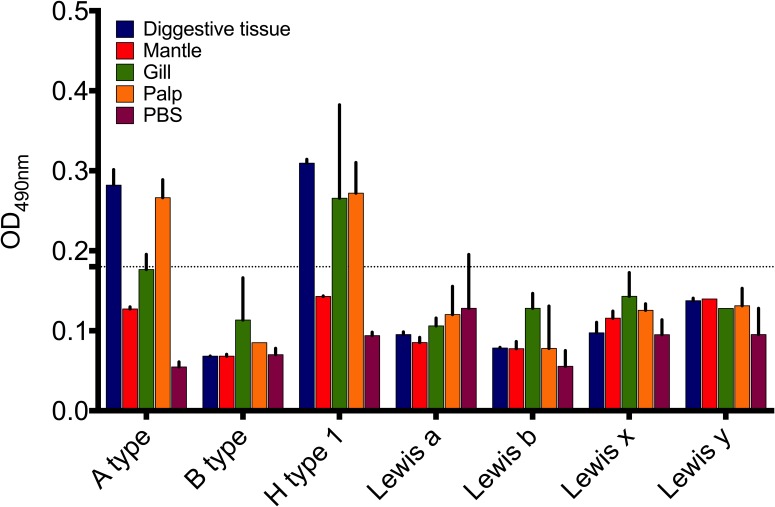
Expression of HBGA-like molecules in digestive tissues, gills, mantle, and palp tissue extracts of Pacific oyster. Data presented as mean (*n* = 5) ± SD.

The HBGA phenotypes present in oyster tissues were examined by eight MAbs which recognize A type, B type, H type 1 and Le^*a,b,x,y*^ HBGA epitopes. The binding cut-off was set at OD_490_ = 0.18, which was three times the value of the PBS control. Only A type and H type 1 carbohydrates were found to be present in the tissues (Figure 2). The A type carbohydrates were detected in the digestive tissues (OD_490_ = 0.28) and palps (OD_490_ = 0.27). Likewise, H type 1 HBGAs were present in the digestive tissues (OD_490_ = 0.31), gills (OD_490_ = 0.26), and palps (OD_490_ = 0.27). None of the other tested HBGAs (B type and Le^*a,b,x,y*^) were detected in the oyster tissue extracts.

### VLP Binding to Saliva Samples

**FIGURE 3:**
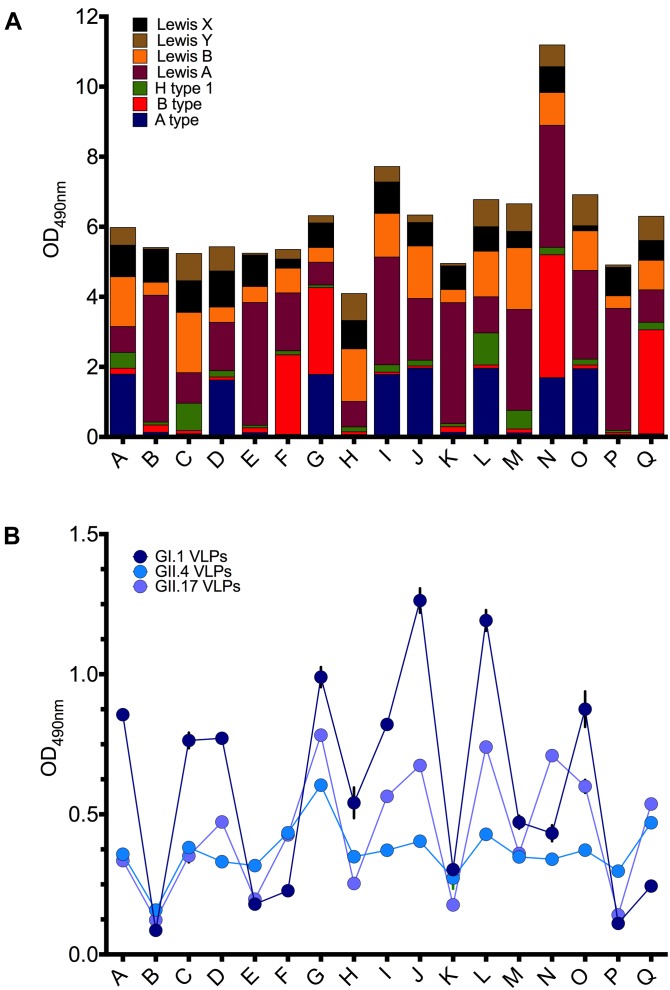
**(A)** Expression of ABH and Lewis antigens in saliva samples (A–Q). **(B)** The binding of GI.1 West Chester VLPs, GII.4 Sydney VLPs, and GII.17 Kawasaki308 VLPs to saliva samples (A–Q). Triplicate assay of 1:50 saliva dilution per well.

To evaluate HBGA binding preferences of the norovirus VLPs in the human host, we performed saliva binding assays using a panel of saliva samples from 17 healthy adult individuals (subject A–Q). Prior to the VLP binding assay, the saliva samples were tested for the ABO, Lewis, and secretor phenotypes using the MAbs. Saliva containing Le^b^ and Le^y^ antigens were defined as secretor positive probes, while the absence of the A, B, H, Le^y^, Le^b^ antigens suggested a non-secretor status. Overall, thirteen samples were secretor positive and four samples were from non-secretors (subjects B, E, K, P) (Figure 3).

The GI.1 West Chester VLPs bound strongly to the saliva probes J and L (OD_490_ > 1.0), containing A/H1/Le^a,b,x,y^ and A/Le^a,b,x,y^ type glycans, respectively (Figure 3A). The moderate binding was also observed for the subjects A (A/H1/Le^a,b,x,y^), C (H1/Le^a,b,x,y^), D (A/Le^a,b,x,y^), G (A/B/Le^a,b,x,y^), I (A/Le^a,b,x,y^), O (A/Le^a,b,x,y^). Weak and no GI.1 VLP binding was observed for saliva samples B, P, E, F, K, which lack A type and H type 1 carbohydrates. Overall, the GI.1 West Chester VLP binding profile matched well-established preferences of GI.1 Norwalk VLPs toward H1, Le^b^ and A type antigens (Harrington et al., 2002; Hutson et al., 2002; Huang et al., 2003; Shirato et al., 2008).

GII.4 VLPs bound to all secretor positive samples, and with an OD_490_ in the 0.4–0.6 range (Figure 3B). Only weak or no binding was observed for the non-secretor saliva samples (B, E, K, P).

The GII.17 VLPs demonstrated the highest signal, with an OD_490_ > 0.67, for subjects G (A/B/Le^a,b,x,y^), J (A/Le^a,b,x,y^), L(A/B/Le^a,b,x,y^), N (A/B/Le^a,b,x,y^) (Figure 3B). Moderate binding of OD_490_ = 0.53–0.56 was observed for the subjects I (A/Le^a,b,x,y^) and Q (B/Le^a,b,x,y^). In contrast, subjects B, K, P, E containing predominantly the Le^a^ and Le^b^ antigens displayed absorbance readings that were below or just above the cut-off limit (OD_490_ < 0.2).

With some exceptions, the saliva binding pattern of GII.17 VLPs closely resembled the binding patterns of GI.1 VLPs (Figure 3B). However, in contrast to GI.1 VLPs, GII.17 VLPs demonstrated a stronger binding for the subjects F, N, Q. These subjects were B positive and A type negative phenotypes, suggesting that GII.17 VLPs recognize H1, Le^b^, A type, but also B type antigens.

### VLP Binding to Synthetic HBGAs

**FIGURE 4:**
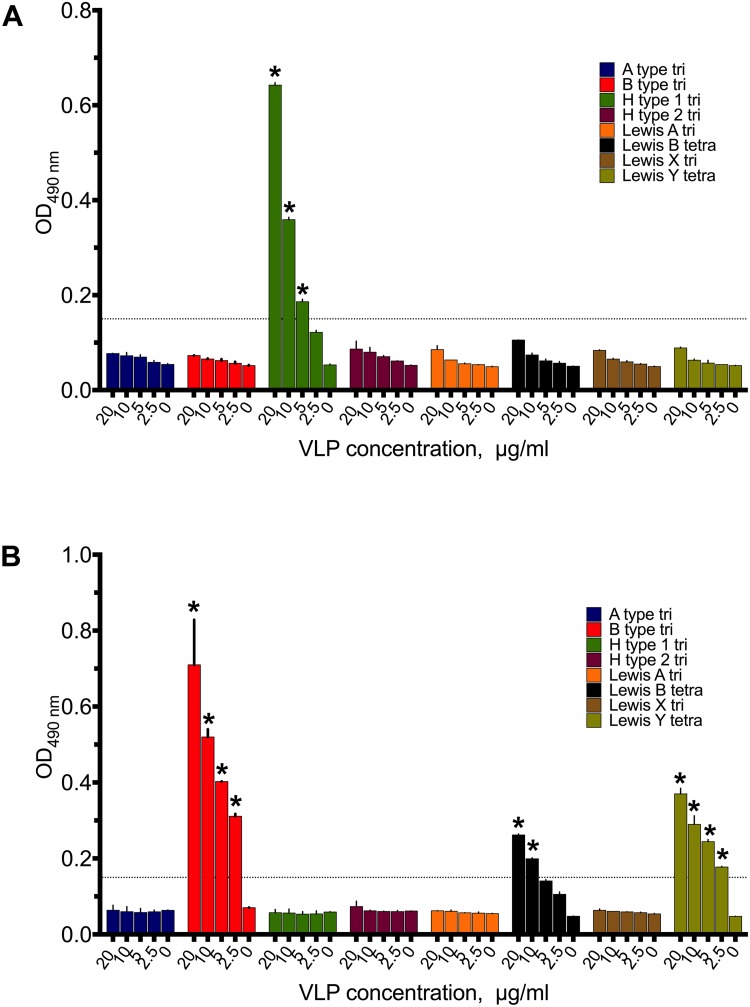
Binding patterns of 20–2.5 μg/ml **(A)** GI.1West Chester VLPs, **(B)** GII.4 Sydney VLPs to PAA-conjugated HBGAs synthetic HBGAs. Triplicate measurements for each VLP concentrations. Asterisks indicate statically significant differences (comparison to PBS control, *p* < 0.05).

Saliva is a complex biofluid containing a mixture of HBGA glycans. To define specificities of GI.1 West Chester, GII.4 Sydney, and the GII.17 Kawasaki308 norovirus VLPs toward individual HBGA types, we further performed a synthetic oligosaccharide-based assay using polyacrylamide-conjugated multivalent HBGAs. As shown in Figure 4A, GI.1 VLPs recognized H type 1 trisaccharides, which was dose-dependent. A higher amounts of GII.4 VLPs bound to B type and lower to Le^y^ and Le^b^ types (Figure 4B). Interestingly, GII.17 VLPs did not bind to tested PAA-conjugated HBGAs, under the studied conditions.

## Discussion

Human noroviruses are genetically and antigenically diverse (Hansman, 2006), with a majority of the human norovirus infections being caused by the GII strains. According to the Noronet report April 2014, approoximately 91% of all norovirus sequences collected worldwide belong to the genogroup GII. 74% of these norovirus sequences were GII.4 variants. GI noroviruses compromised only approximately 9% of all outbreaks. Most of the norovirus genotypes which infect humans have also been found in oyster-related outbreak samples. However, the GI and GII proportions are significantly different in oyster-related outbreaks than in non-oyster related outbreaks. In oyster-related outbreaks, only 66% of all norovirus sequences found belonged to the GII strains (Noronet, 2014)^1^. Moreover, the dominant GII.4 strains only accounted for 30% of the GII sequences reported. This shift could have been driven by the more frequent occurrence of GI strains in coastal waters. However, influent and effluent waters still contain higher concentrations of GII noroviruses (Flannery et al., 2012), indicating that oysters are also exposed to higher GII concentrations. Consequently, GI noroviruses must be enriched in oyster tissues, where selection and persistence is dependent on specific molecular interactions between the virus and the oyster. Such specific viral enrichment might be mechanistically similar to HBGA-norovirus binding in humans (Tian et al., 2006, 2007; Maalouf et al., 2011).

Histo-blood group antigens located on epithelial cells of the human gastrointestinal tract are receptors or co-receptors for human noroviruses. Certain HBGA types are also expressed in oyster tissues. Tian et al. (2007) detected A type-like and H type-like HBGAs in the gastrointestinal tissues of *Crassostrea virginica, Crassostrea gigas*, and *Crassostrea sikamea*. Similarly, in our study we were able to demonstrate that both A type and H type 1 were present in the digestive tissues of *Crassostrea gigas.* Additionally, we demonstrated that the palps also contained these HBGAs, while gills and mantle showed undetectable levels of any HBGA types.

Norovirus interaction with HBGAs has been shown to occur via the protruding (P) domain of the major capsid VP1 protein (Cao et al., 2007; Choi et al., 2008; Hansman et al., 2011). Distinct structural features of the P domains from different norovirus strains define their binding preferences toward specific HBGA types. A summary of the HBGA binding preferences of the GI.1 West Chester, the pandemic GII.4 Sydney, and the epidemic GII.17 Kawasaki308 norovirus VLPs is presented in Table 1. We observed that GI.1 VLPs used in this study readily reacted with A type, AB type, and H type, but not with B type saliva. Moreover, these GI.1 VLPs also bound to synthetic PAA conjugated H type 1 HBGAs. This matches previous results of another GI.1 strain, which demonstrated that the prototype GI.1 Norwalk noroviruses preferentially recognize A-type and H-type 1 HBGAs, and might also bind Le^b^ antigens (Harrington et al., 2002; Hutson et al., 2002; Huang et al., 2003; Shirato et al., 2008). As was expected from its HBGA binding capabilities, we found that GI.1 VLPs reacted with digestive tissues and palps of *Crassostrea gigas*, which predominantly contained A type and H type HBGAs. This further supports previous observations obtained from oysters (Maalouf et al., 2011).

**Table 1:** Summary of the HBGA binding preferences combined from saliva and synthetic HBGA assays.

	A type	B type	H type 1	Le^a^	Le^b^	Le^x^	Le^y^
GI.1 West Chester	+	-	+	-	+	-	-
GII.4 Sydney	-	+	+	-	+	-	+
GII.17 Kawasaki308	+	+	+	-	+	-	-


The most prevalent GII.4 noroviruses seem to possess a broad and long-standing HBGA binding profile. Evolutionary studies revealed a highly conserved HBGA binding site in the GII.4 norovirus strains isolated throughout the 1974–2012 period (Bok et al., 2009). With the exception of the structural studies (Singh et al., 2015; Morozov et al., 2018), the HBGA binding preferences of the most virulent GII.4 Sydney strain were largely unexplored. Here, we demonstrated that the GII.4 Sydney VLPs interacted equally well to secretor and non-secretor saliva samples. Non-secretors do not express H1 and Lewis-b epitopes, and hence the observed binding could be due to Lewis-y or H2 interference. In the synthetic HBGAs assays, the GII.4 Sydney VLPs reacted with B type trisaccharide-PAA, Le^b^ type tetrasaccharide-PAA, Le^y^ type tetrasaccharide-PAA conjugates. However, the latter results have to be treated with caution, due to that fact that the short carbohydrate chains are attached to a polymer linker, and are therefore less accessible for the bulky VLP structure. Our parallel work, using carbohydrate mixtures from breast milk (human milk oligosaccharides, HMOs), revealed the specificity of GII.4 Sydney VLPs toward longer saccharides containing 4–15 sugar units and the terminal blood group H type 1 or Lewis-b epitopes (Hanisch et al., 2018). Together these results suggest that GII.4 Sydney VLPs can recognize a broad spectrum of carbohydrates, including Le^b^, H type 1, Le^y^, and B type blood group antigens, although the affinities to each specific HBGA type remain to be elucidated. In this study, GII.4 Sydney bound well to all the investigated oyster tissues. In case of the digestive tissues this can be explained by recognition of the H type 1 HBGAs, while the binding to the mantle and gill tissues can most likely be explained by the presence of non-HBGA carbohydrates, as it was previously observed for GII.4 Houston VLPs (Maalouf et al., 2011).

Enhanced HBGA binding capabilities have been put forward as a factor to explain the rapid emergence of the GII.17 strains (Chan et al., 2015b). However, in our synthetic HBGA assay, only low concentrations of GII.17 Kawasaki308 VLPs associated with the sugars. As previously mentioned, this may have been due to steric effects, the affinity of GII.17 Kawasaki308 to short-chain HBGA-containing PAAs used on our plates was likely lower compared to GII.4, and GI.1 strains. GII.17 Kawasaki308 VLPs could nevertheless interact with longer saccharides and with glycoproteins present in saliva of secretor-positive individuals. Its binding patterns closely resembled those of GI.1 VLPs, but showed additional signals in B type subjects, which supports its enhanced binding capabilities. We observed no binding of GII.17 Kawasaki308 to the oyster tissues extracts, therefore we assumed that GII.17 Kawasaki308 exhibited low affinity interactions with endogenous blood group active oligosaccharides (A type, B type, H type, Le^b^ blood group epitopes), and,thus, just weakly attach to the HBGA epitopes expressed in the digestive tissues of pacific oysters.

Considering these binding assay results it is surprising that oysters can be contaminated with GII.17 noroviruses (Shin et al., 2013; Pu et al., 2016; Rasmussen et al., 2016; La Rosa et al., 2017). One explanation could be that the epidemic GII.17 noroviruses do not actively accumulate through tissue binding in oysters and only use oysters as a short term transmission vector via passive accumulation during filter feeding. This might also apply to other norovirus genotypes detected in oyster related outbreaks. Oyster only express a limited array of HBGA types that, in some cases, cannot be recognized by every norovirus strain. It is therefore suggested, that many noroviruses can interact with tissues only non-specifically, while other noroviruses, i.e., GI.1, can be efficiently accumulated within oysters. Such binding-dependent accumulation could explain the shift toward the increased presence of GI genotypes in oysters (Yu et al., 2015). Alternatively, other unidentified proteins or, as it was shown for the GII.4 Houston strain, non-HBGA glycans could act as selective ligands for the norovirus accumulations in oysters (Maalouf et al., 2010). These molecules or a larger variety of HBGAs could also be provided by the diverse microbiome of the oyster digestive tract (Lokmer et al., 2016) that might therefore, serve as a substrate for norovirus recognition and accumulation (Miura et al., 2013; Li et al., 2015).

From the combination of binding assays applied in this study, we conclude that the pandemic GII.4 Sydney norovirus strains are likely to employ H type 1-mediated interactions to accumulate within oysters, which could potentially lead to long-term transmission. The epidemic GII.17 noroviruses, on the other hand are likely to have low persistence in oysters and transmission is suggested to be short-term, when high environmental concentration of these viruses is found. Understanding these strain specific characteristics of persistence will allow the prediction of norovirus epidemiology, which is vital for the development of control measures.

## Ethics Statement

This study was carried out in accordance with the recommendations of the ethics committee of the Medical Faculty of Mannheim, Heidelberg University, 2017-528N-MA, with written informed consent from all subjects. All subjects gave written informed consent in accordance with the Declaration of Helsinki. The protocol was approved by the ethics committee of the Medical Faculty of Mannheim, Heidelberg University, 2017-528N-MA.

## Author Contributions

VM, KMW, and HS conceptualized the idea. VM, F-GH, KMW, and HS analyzed the data and wrote the original draft. VM performed the experiments. All authors had read and approved the final version of the manuscript.

## Conflict of Interest Statement

The authors declare that the research was conducted in the absence of any commercial or financial relationships that could be construed as a potential conflict of interest.

## Footnotes

1 https://www.rivm.nl/en/Documents_and_publications/Common_and_Present/Publications/Centre_for_Infectious_Disease_Control/Noronet_updates/Noronet_update_april_2014
