# Supplementary material for: Pandemic GII.4 Sydney and Epidemic GII.17 Kawasaki308 Noroviruses Display Distinct Specificities for Histo-Blood Group Antigens Leading to Different Transmission Vector Dynamics in Pacific Oysters
Source: Front Microbiol. 2018 Nov 27;9:2826. doi: 10.3389/fmicb.2018.02826 (PMC6278567; doi:10.3389/fmicb.2018.02826)
Supplement: Supplementary file 1 [file Data_Sheet_1.docx]

Supplementary Material

Pandemic GII.4 Sydney and Epidemic GII.17 Kawasaki308 Noroviruses Display Distinct Specificities for Histo-Blood Group Antigens leading to different transmission vector dynamics in Pacific oysters

Vasily Morozov^*^, Franz-Georg Hanisch, K. Mathias Wegner and Horst Schroten

*** Correspondence:** Vasily Morozov: [vasily.morozov@medma.uni-heidelberg.de](mailto:vasily.morozov@medma.uni-heidelberg.de)


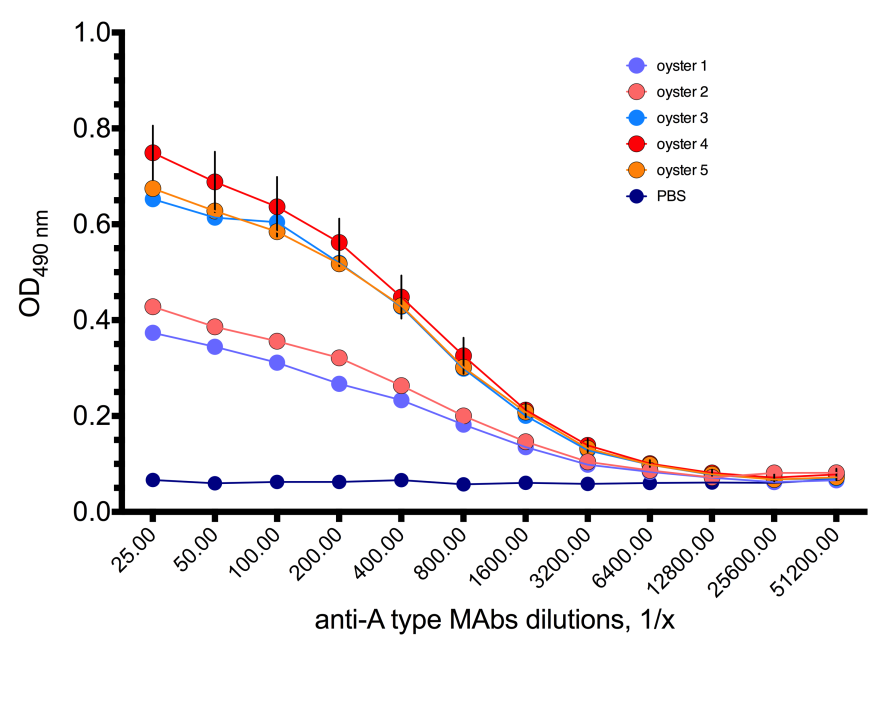


**Figure S1.** Expression of A type HBGA in digestive tissues of individual oysters measured by EIA as described in Materials and Methods.


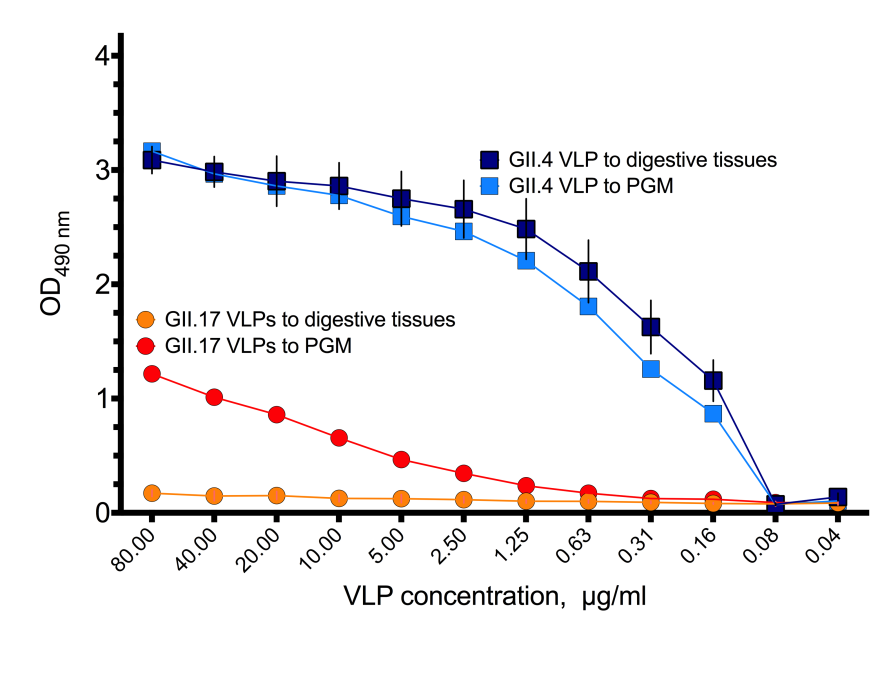


**Figure S2.** The binding of 80 – 0.04 µg/ml GII.17 Kawasaki308 and GII.4 Sydney VLPs to PGM and digestive tissues of oysters.


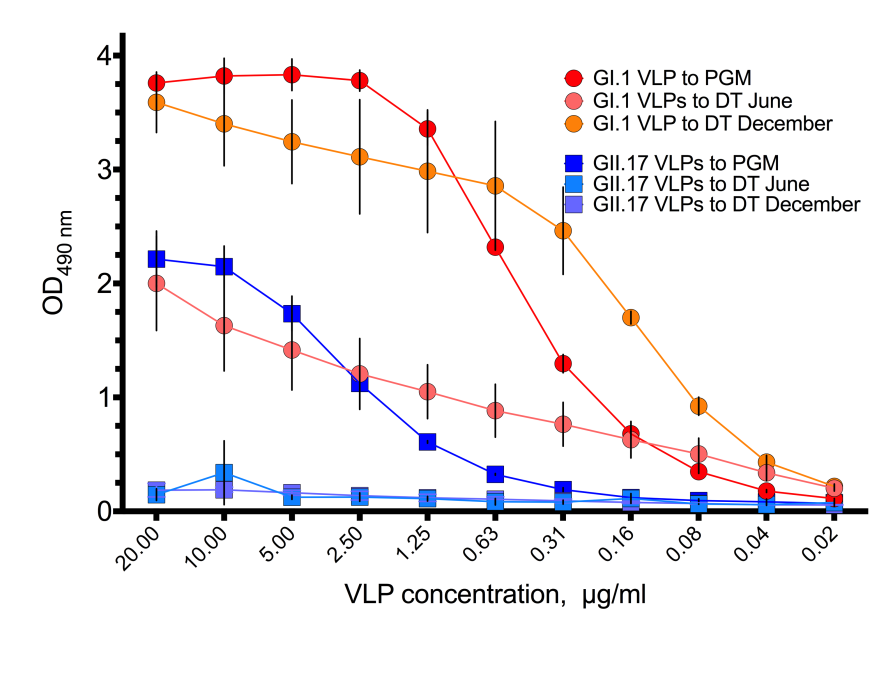


**Figure S3.** The binding of 20 – 0.04 µg/ml GII.17 Kawasaki308 and GI.1 Wester Chester VLPs to PGM and digestive tissues of oyster collected in December 2016 and June 2017.
